# 

**Published:** 1834-10-01

**Authors:** 


### CONTENTS

OF THE

### MEDICO-CHIRURGICAL REVIEW,

No. XLII. OCTOBER 1, 1834.

[BEING No. II. OF A DECENNIAL SERIES.]

### REVIEWS.

I.

Leçons Orales de Clinique Chirurgicale, faites à l'Hôtel Dieu de Paris. Par M. Dupuytren .. .. .. .. .. .. .. .. .. .. .. .. .. .. .. 289

II.

Pathological and Surgical Observations on Diseases of the Joints. By B. C. Brodie, V.P.R.S... .. .. .. .. .. .. .. .. .. .. .. .. .. 331

III.

Physiologie Végétale. Par A. P. de Caudolle .. .. .. .. .. .. .. .. .. .. 353

IV.

An Inquiry into the Nature of Sleep and Death, &c. By A. P. W. Philip, M.D.. 359

V.

A New System of Organic Chemistry. By F. V. Raspail. Translated by W. Henderson, M.D. .. .. .. .. .. .. .. .. .. .. .. .. .. .. .. 365

VI.

A Treatise on the Diseases and Injuries of the Spinal Nerves. By Jos. Swan, Esq. 371

VII.

A Treatise on the Circulation of the Blood. By J. F. Handley, Esq. .. .. .. 390

VIII.

A Treatise on the Diseases of the Skin. By P. Rayer, D.M.P. Translated by W. B. Dickinson, Esq. .. .. .. .. .. .. .. .. .. .. .. .. .. .. .. .. 391

IX.

The Inaugural Dissertation of C. F. J. Bellingeri .. .. .. .. .. .. .. .. 413

X.

WORKS ON PATHOLOGICAL ANATOMY:

1. Principles and Illustrations of Morbid Anatomy. By J. Hope, M.D Part XII.
2. Illustrations of the Elementary Forms of Disease. By Robt. Carswell, M.D. Fasciculus VI. .. .. .. .. .. .. .. .. .. .. .. .. .. .. 424

### PERISCOPE.

PART I.

### Spirit of the English Periodicals, and Notices of English Medical Literature.

1. Influence of Bodily Position on the Pulse. By T. R. Blackley, A.B.. .. .. 433

2. Danger of Ergot of Rye in Large Doses.. .. .. .. .. .. .. .. .. .. .. 435

3. On Scrofula. By J. Eager, M.D. .. .. .. .. .. .. .. .. .. .. .. .. 435

4. Anomalous Tumor in the Right Hypochondrium.. .. .. .. .. .. .. .. 438

5. The Rev. Thos. Everest on Homœopathy .. .. .. .. .. .. .. .. 439

6. Somnambulism. Case of Jane Ryder .. .. .. .. .. .. .. .. .. .. .. 440
7. Nitrate of Silver in Epidemic Cholera. By C. Lever, M.D. .. .. .. .. 444
8. Observations on Internal Aneurism. By Professor Harrison, of Dublin.. .. 445
Comments of Dr. Stokes .. .. .. .. .. .. .. .. .. .. .. .. .. .. .. 448
9. On Manual Assistance in Abortion. By Mr. Wainwright, of Liverpool .. .. 448
10. Dr. James Johnson's Case of Crackling of the Muscles .. .. .. .. .. .. 452
11. Case of Hydatids of the Kidney voided by the Urethra .. .. .. .. .. .. 453
12. Mr. Tyrrell on Catarrhal and Catarrho-Rheumatic Ophthalmia .. .. .. 454
A. Catarrhal Ophthalmia .. .. .. .. .. .. .. .. .. .. .. .. .. .. .. 454
B. Catarrho-Rheumatic Ophthalmia .. .. .. .. .. .. .. .. .. .. .. 455
C. Treatment .. .. .. .. .. .. .. .. .. .. .. .. .. .. .. .. .. .. 456
13. Researches of Dr. Stokes on the Diagnosis and Pathology of Aneurism .. .. 458
A. Aneurism of the Hepatic Artery .. .. .. .. .. .. .. .. .. .. .. 458
B. Aneurism of the Innominata .. .. .. .. .. .. .. .. .. .. .. .. 459
C. Rupture of the Ascending Aorta .. .. .. .. .. .. .. .. .. .. .. 461
D. Aneurism of the Thoracic Aorta, recognized during Life .. .. .. .. .. 461
14. A Practical Treatise on Medical Jurisprudence. By J. Chitty, Esq. Barrister 462
15. Mr. Thurnam's Dissections of the Otic Ganglion .. .. .. .. .. .. .. .. 465
16. An Essay on the Relation of the Theory of Morals to Insanity. By T. Mayo, M.D. .. .. .. .. .. .. .. .. .. .. .. .. .. .. .. .. .. .. .. .. 466
17. Dr. Forbes on the Climate of the Land's End .. .. .. .. .. .. .. .. 468
18. Mr. Sandwith on Hooping-Cough .. .. .. .. .. .. .. .. .. .. .. .. .. 471
19. The Morbus Oryzeus, and Atrocities of Ricemen.. .. .. .. .. .. .. .. 471
20. On Mr. Stafford's Plan of perforating Strictures .. .. .. .. .. .. .. .. 473
21. Dr. A. T. Thompson on Ioduret and Hydriodate of Iron .. .. .. .. .. .. 476
22. A Dictionary of Terms employed by the French in Anatomy, Physiology, &c. By Shirley Palmer, M.D.. .. .. .. .. .. .. .. .. .. .. .. .. .. .. 477
23. Mr. Guthrie's Operation of tying the Common Iliac Artery .. .. .. .. 478
Remarks upon the Case .. .. .. .. .. .. .. .. .. .. .. .. .. .. .. 481
24. Reclamation of Valentine Mott, with Reference to the Operation of tying the Common Iliac Artery with Success .. .. .. .. .. .. .. .. .. .. .. 483
25. Case of Fracture of the Skull, with Depression. By Mr. Cotton, of Bagshot.. 483
26. The Cholera .. .. .. .. .. .. .. .. .. .. .. .. .. .. .. .. .. .. 484
A. Innumerable Specifics for it .. .. .. .. .. .. .. .. .. .. .. .. 485
B. The Treatment by Emetics of Salt and Water.. .. .. .. .. .. .. .. 486
C. The Suggestions of the Editors.. .. .. .. .. .. .. .. .. .. .. .. 487
27. Case of Chronic Enlargement of the Clitoris. Treated by Mr. Edward, of Forfar .. .. .. .. .. .. .. .. .. .. .. .. .. .. .. .. .. .. .. .. 489
28. Literature of the Quarter.. .. .. .. .. .. .. .. .. .. .. .. .. .. .. 489
1. Illustrations of the Effects of Poisons. By Dr. Roupell and Mr. M'Whinnie 490
A. Effect of Concentrated Sulphuric Acid .. .. .. .. .. .. .. .. .. 490
B. Effect of Oxalic Acid .. .. .. .. .. .. .. .. .. .. .. .. .. .. 490
C. Effect of Corrosive Sublimate .. .. .. .. .. .. .. .. .. .. .. 491
D. Effect of Alkohol .. .. .. .. .. .. .. .. .. .. .. .. .. .. .. 491
2. Fragmenta de Viribus Medicamentorum Positivis. A. Saumele Hahnemann. Edidit F. F. Quin, M.D. .. .. .. .. .. .. .. .. .. .. .. .. 492
3. The Principles of Diagnosis. By Marshall Hall, M.D. Vol. I. 2d Edition, 493
4. A Demonstration of the Nerves of the Human Body. Quarto. By Jos. Swan, Esq. .. .. .. .. .. .. .. .. .. .. .. .. .. .. .. .. .. .. 494

5. An Essay on the Deaf and Dumb. By J. Curtis, Esq. .. .. .. .. .. .. 495
6. A Practical Treatise on the Lepra Vulgaris. By Edward Beck, M.D. .. 495

PART II.

### Spirit of the Foreign Periodicals.

29. On the Operation of Staphyloraphy.. .. .. .. .. .. .. .. .. .. .. .. 495
30. On the Curative Effects of Abstinence .. .. .. .. .. .. .. .. .. .. 498
31. On the Operation of Pelviotomy .. .. .. .. .. .. .. .. .. .. .. .. 500
32. Removal of Calculi from the Perineum.. .. .. .. .. .. .. .. .. .. 501
33. Disease of the Cerebellum, and curious Disturbance of the Muscular Powers.. 501
34. Hypertrophy of the Muscular Coat of the Stomach .. .. .. .. .. .. .. 502
35. Inflammation of the Membranes of the Ovum .. .. .. .. .. .. .. .. 502
36. Case of Fatal Opisthotonos .. .. .. .. .. .. .. .. .. .. .. .. .. 503
37. Cancerous Ulceration of the Nose, treated with Creosote .. .. .. .. .. .. 505
38. On the Employment of Soot as a Substitute for Creosote .. .. .. .. .. .. 506
Cases and Experiments .. .. .. .. .. .. .. .. .. .. .. .. .. .. 506
39. Lotions of Corrosive Sublimate for Ophthalmia .. .. .. .. .. .. .. .. 509
40. On the Use of Chloruret of Lime in Blennorrhagia .. .. .. .. .. .. .. 510
41. On the Section of the Tendo Achillis in Cases of Club-Foot .. .. .. .. .. 510
42. Spontaneous Dry Gangrene cured by Bleeding .. .. .. .. .. .. .. .. 512
43. M. Dupuytren on Hydrocele of the Neck .. .. .. .. .. .. .. .. .. .. 513
44. On the Paralysis induced by the Preparations of Lead .. .. .. .. .. .. 514
45. Literary Curiosities .. .. .. .. .. .. .. .. .. .. .. .. .. .. .. .. 515
46. Chemical Examination of Spots caused by Blood and Semen on Linen .. .. 516
47. Analytical Epitome of the Second and Third Sections of Cayol's Traité des Maladies Cancéreuses .. .. .. .. .. .. .. .. .. .. .. .. .. 517
A. External Remedies employed for Cancer .. .. .. .. .. .. .. .. .. 517
B. Internal Remedies so employed.. .. .. .. .. .. .. .. .. .. .. .. 520
C. Salts and other Mineral Substances .. .. .. .. .. .. .. .. .. .. 521
D. Different Empirical Remedies .. .. .. .. .. .. .. .. .. .. .. .. 522
48. Vaginal Discharges suspected to arise from Violation—in very young females, 524
49. On certain Mental Illusions .. .. .. .. .. .. .. .. .. .. .. .. .. 524
50. Further Particulars respecting the Properties of Creosote.. .. .. .. .. .. 527
51. On Hypertrophy of the Mammæ .. .. .. .. .. .. .. .. .. .. .. .. 528

PART III.

### Clinical Review and Hospital Reports.

St. George's Hospital.

52. A. Clinical Remarks on Ununited Fractures. By Mr. Brodie .. .. .. .. 529
B. Clinical Remarks on some Cases of Disease of the Urinary Organs. By Cæsar Hawkins, Esq. .. .. .. .. .. .. .. .. .. .. .. .. .. 530
C. Report on Hernia, from the Case-Book of Mr. Hawkins .. .. .. .. .. 533
1. Strangulated Hernia—early Operation .. .. .. .. .. .. .. .. .. 533
2. Incarcerated Omental Hernia .. .. .. .. .. .. .. .. .. .. .. 534
3. Wearing a Truss on an Irreducible Hernia .. .. .. .. .. .. .. 535
4. Rupture of Intestine by a Blow upon a Hernia .. .. .. .. .. .. 535
5. Hernia produced by a Blow .. .. .. .. .. .. .. .. .. .. .. 536
6. Treatment after the Operation for Hernia .. .. .. .. .. .. .. 537
7. Operation followed by Prostration .. .. .. .. .. .. .. .. .. 537
8. Operation twice performed—sloughing of the Intestine.. .. .. .. 539

LONDON DISPENSARY.

53. On the best mode of treating Mammary Abscess.. .. .. .. .. .. .. .. 540

MEATH HOSPITAL.

54. Dr. Stokes on the Neuroses—Cerebritis—Phrenology.. .. .. .. .. .. .. 542

MIDDLESEX HOSPITAL.

55. Cases of Poisoning by Barytes and the Mineral Acids .. .. .. .. .. .. 548
1. Poisoning by Carbonate of Barytes .. .. .. .. .. .. .. .. .. .. 549
2. Poisoning by Nitric Acid.. .. .. .. .. .. .. .. .. .. .. .. .. 549
3. Poisoning by Sulphuric Acid .. .. .. .. .. .. .. .. .. .. .. .. 549

CITY OF LIMERICK INFIRMARY.

56. Surgical Report of Mr. Kane .. .. .. .. .. .. .. .. .. .. .. .. .. 550

RICHMOND SURGICAL HOSPITAL.

57. Dr. O'Beirne on Hydrocele of the Neck.. .. .. .. .. .. .. .. .. .. 554

MEATH HOSPITAL.

58. Clinical Remarks of Dr. Graves on various Diseases .. .. .. .. .. .. .. 557
1. On simple Pneumo-thorax .. .. .. .. .. .. .. .. .. .. .. .. 558
2. Bruit de Soufflet of the Heart in Pneumonia .. .. .. .. .. .. .. 559
3. Symmetrical Erysipelas .. .. .. .. .. .. .. .. .. .. .. .. .. 559
4. On the best Mode of administering Calomel in Acute Inflammation, .. 559
5. Spontaneous Cure of Chronic Ascites .. .. .. .. .. .. .. .. .. .. 560
6. Diffuse Inflammation fatal from Effusion into the Chest .. .. .. .. .. 560
7. Loss of the Sense of Smelling.. .. .. .. .. .. .. .. .. .. .. .. 561
8. Carbonate of Ammonia in the Urine .. .. .. .. .. .. .. .. .. .. 561
9. Albuminous Urine in Dropsy .. .. .. .. .. .. .. .. .. .. .. .. 562
10. Diabetes Insipidus .. .. .. .. .. .. .. .. .. .. .. .. .. .. 563

MANCHESTER ROYAL INFIRMARY.

59. Dr. Carbutt's Clinical Lectures .. .. .. .. .. .. .. .. .. .. .. .. 563

**Miscellanies.**

60. The North-West London Self-supporting Dispensary .. .. .. .. .. .. .. 567

61. Last Curriculum of the College of Surgeons.. .. .. .. .. .. .. .. .. 569

Circular to Lecturers.. .. .. .. .. .. .. .. .. .. .. .. .. .. .. .. 570

62. Provincial Medical Schools .. .. .. .. .. .. .. .. .. .. .. .. .. .. 571

63. Baronetcy of Mr. Brodie .. .. .. .. .. .. .. .. .. .. .. .. .. .. 573

Bibliographical Record .. .. .. .. .. .. .. .. .. .. .. .. .. .. .. 574

